# Circulating Tumour DNA Is an Independent Prognostic Biomarker for Survival in Metastatic *BRAF* or *NRAS*-Mutated Melanoma Patients

**DOI:** 10.3390/cancers12071871

**Published:** 2020-07-11

**Authors:** Guillaume Herbreteau, Audrey Vallée, Anne-Chantal Knol, Sandrine Théoleyre, Gaelle Quéreux, Cécile Frénard, Emilie Varey, Paul Hofman, Amir Khammari, Brigitte Dréno, Marc G. Denis

**Affiliations:** 1 Department of Biochemistry, CHU Nantes, 44093 Nantes, France; guillaume.herbreteau@chu-nantes.fr (G.H.); audrey.vallee@chu-nantes.fr (A.V.); sandrine.charpentier@chu-nantes.fr (S.T.); 2 Centre de Recherche en Cancérologie et Immunologie Nantes-Angers (CRCINA) Inserm 1232, Centre Hospitalier Universitaire de Nantes (CHU Nantes), 44093 Nantes, France; annechantal.knol@chu-nantes.fr (A.-C.K.); gaelle.quereux@chu-nantes.fr (G.Q.); cecile.frenard@chu-nantes.fr (C.F.); emilie.varey@chu-nantes.fr (E.V.); amir.khammari@chu-nantes.fr (A.K.); brigitte.dreno@atlanmed.fr (B.D.); 3 Department of Dermato Cancerology, CHU Nantes, 44093 Nantes, France; 4 Centre d’investigation Clinique (CIC) 1413, CHU Nantes, 44093 Nantes, France; 5 Laboratory of Clinical and Experimental Pathology, Pasteur Hospital, University Côte d’Azur, 06000 Nice, France; HOFMAN.P@chu-nice.fr; 6 Institut de Recherche sur le Cancer et le Vieillissement (IRCAN) Inserm 1081/the National Center for Scientific Research (CNRS) 7284, Antoine Lacassagne Center, 06002 Nice, France

**Keywords:** melanoma, circulating tumour DNA, BRAF, NRAS, mutation, prognosis

## Abstract

Circulating tumour DNA (ctDNA) can be used to identify gene alterations. The purpose of this study was to determine whether the detection of ctDNA, based on the identification of *BRAF* and *NRAS* mutations before systemic treatment initiation, was associated with the prognosis of metastatic melanoma. In total, 68 *BRAF* or *NRAS*-mutated stage IV or unresectable stage III metastatic cutaneous melanoma patients were included and tested for the presence of *BRAF* and *NRAS* mutations in circulating DNA before treatment initiation, using the Cobas BRAF/NRAS Mutation Test (Roche). The expected mutation was detected in the plasma of 34/68 patients (50% sensitivity). ctDNA detection was associated with AJCC stage, along with the number and nature of metastases. ctDNA was less frequently detected in *NRAS*-mutated than in *BRAF*-mutated melanoma (36% and 66%, respectively). At initiation of first-line treatment, ctDNA detection was associated with poor prognosis in Progression Free Survival (PFS) and Overall Survival (OS) in univariate analysis (log-rank: *p* = 0.002 and *p* < 0.0001, respectively). In multivariate analysis, ctDNA detection was an independent factor of poor prognosis in OS, after adjustment for AJCC stage, number and nature of metastases and gender (HR = 4.384; 95% CI: (1.308; 14.699); *p* = 0.017).

## 1. Introduction

In the era of precision medicine, accurate assessment of cancer prognosis is essential to stratify risks and determine the best treatment option. The classification of the American Joint Committee on Cancer (AJCC) guides the management of cutaneous melanoma. At AJCC stages I and II (localised extension), treatment is only surgical, with an excellent prognosis. At stage III, the locoregional cutaneous and lymph node extension marks a turning point in the disease. The presence of a positive sentinel node leads to a lymph node dissection of the invaded area, and subsequently, to adjuvant treatment with anti-PD1 immunotherapy or with BRAF inhibitors (BRAFi), depending on whether the tumour harbours a *BRAF* codon 600 mutation. At the distant metastatic stage (AJCC stage IV), the prognosis drops drastically, with a 5-year overall survival of 20%. As a first line of treatment, BRAF inhibitors are indicated in combination with MEK inhibitors (MEKi) for patients with *BRAF*-mutated metastatic melanoma (40% of cases). Immune checkpoint inhibitors (anti-PD1, anti-CTLA4 or a combination of both) constitute a therapeutic alternative, both in *BRAF*-wildtype and *BRAF*-mutated patients.

However, despite the performance of the AJCC classification, the prognosis of patients within each risk group remains heterogeneous. New prognostic factors are needed to assess the risk for each patient and to determine the best treatment option, especially among the new and recently validated therapeutic options in stage IV melanoma, such as: (i) single anti-PD1 immunotherapy versus combination with anti-CTLA4 immunotherapy [1]; (ii) first-line targeted therapy versus first-line immunotherapy in *BRAF*-mutated melanoma [2,3].

Circulating tumour DNA (ctDNA), released into the bloodstream by cancer cells, is a source of tumour DNA complementary to or even alternative to tumour tissue DNA for many cancers. ctDNA is commonly used to identify mutations in the *EGFR* gene in non-small cell lung cancer (NSCLC), when tumour tissue cannot be sampled or when its analysis is not contributive [4,5]. Although melanoma tumour tissue is more often accessible, ctDNA can be used to quickly identify theranostic *BRAF* mutations, without going through the various preanalytical steps commonly performed on tumour tissue (formalin fixation, paraffin embedding, preparation of thick sections) [6]. 

Several studies have suggested that the detection of ctDNA, i.e., the identification of genetic alterations in circulating cell-free DNA, may be a prognostic factor for metastatic melanoma. In a previous study carried out on baseline detection of *BRAF* codon 600 mutations by Amplification-Refractory Mutation System (ARMS) qPCR in patients with metastatic melanoma harbouring a *BRAF* mutation in tumour tissue, we identified the mutation in 29 out of the 38 patients tested [7]. ctDNA detection was significantly associated with an unfavourable prognosis in overall survival (OS), and was also correlated with AJCC stage, number of metastatic sites and serum LDH activity. 

Mutations in the *BRAF* and *NRAS* genes are the most frequent alterations described in cutaneous melanomas, with a prevalence of ~50% and ~25%, respectively [8,9,10,11]. Therefore, *BRAF* or *NRAS*-mutated melanomas represent the vast majority of patients. This study aimed to determine whether the detection of ctDNA, based on the rapid identification of common *BRAF* and *NRAS* mutations before systemic treatment initiation, was associated with the prognosis of metastatic melanoma, in an intention-to-treat setting.

## 2. Results

### 2.1. Patient Characteristics

In total, 68 patients starting a systemic treatment (immunotherapy or targeted therapy) at Nantes University Hospital for a *BRAF* or *NRAS*-mutated stage IV or unresectable stage III metastatic cutaneous melanoma were included. Patient characteristics are summarised in Table 1. The cohort included 31 women and 37 men, with an average age of 62.0 years (median 65.0 years; min 20.6 years; max 95.2 years). A total of 32 patients had a *BRAF*-mutated melanoma and 36 had an *NRAS* mutation. All *NRAS*-mutated patients were treated with immunotherapy. Among the *BRAF*-mutated patients, 25/32 (78%) were treated with a targeted therapy (8 with BRAFi, 17 with a BRAFi/MEKi combination) and 7/32 (22%) were treated with immunotherapy (35 with anti-PD1, 2 with anti-CTLA4 and 6 with an anti-PD1/anti-CTLA4 combination). 

Most patient characteristics did not differ significantly between *BRAF* and *NRAS*-mutated patients, with the exception of gender: *NRAS* mutations were more frequent in women (*BRAF*-mutated: 8/31 (26%); *NRAS*-mutated: 23/31 (74%)), while men were mostly *BRAF*-mutated (*BRAF*-mutated: 24/37 (65%); *NRAS*-mutated: 13/37 (35%); *p* = 0.002).

### 2.2. CtDNA Detection

The plasma samples taken before treatment initiation were extracted and analysed with the Cobas system. *BRAF* or *NRAS* mutations previously identified in tumour tissues were found in 34 out of 68 patients, representing an overall 50% sensitivity between tissue analyses and circulating DNA analysis on the Cobas system. 

ctDNA detectability was compared to patient characteristics in a subgroup analysis (Table 2). ctDNA detection was associated with stage (detectable ctDNA in 30/47 (64%) of stage IV melanomas vs. 4/21 (19%) of unresectable stage III melanomas; *p* = 0.001), number of metastases (means: 3.9 and 3.3, whether ctDNA was detectable or undetectable, respectively; *p* = 0.002) and the presence of pulmonary, abdominal, bone and lymph node metastases. Furthermore, ctDNA was less detected in *NRAS*-mutated patients (*NRAS* mutations detectable in 13/36 patients (36%) vs. *BRAF* mutations detectable in 21/32 patients (66%); *p* = 0.028) and in women (ctDNA detectable in 9/31 women (29%) vs. 25/37 men (68%); *p* = 0.003). The lower detectability of ctDNA in women was not linked to the higher representation of *NRAS* mutations in this subgroup, in multivariate analysis (logit: *p* = 0.013).

### 2.3. Diagnostic Performance of the Cobas System

In this study, *NRAS* mutations were less frequently detected in circulating DNA by the Cobas system than *BRAF* mutations. An evaluation of the diagnostic performance of the Cobas system, anticipated in the study protocol, was performed halfway through the inclusion period. The results obtained by the Cobas analysis were compared to those obtained by dPCR on the first 31 patients included (Table 3).

There was a strong agreement between the results of the Cobas analysis and those obtained by dPCR (Cohen’s κ = 0.71). Mutations identified in tumour tissue were found in circulating DNA with both techniques for 18 patients, while ctDNA was undetectable with both techniques for nine patients. For two patients, a *BRAF* mutation identified in the tumour tissue was found in circulating DNA by dPCR, but was not identified by the Cobas analysis. In both cases, these *BRAF* V600E mutations were very poorly represented in the sample (8 copies/mL of plasma, allelic frequency < 0.002%). In contrast, ctDNA was detected by Cobas analysis in two dPCR-negative patients, including one patient with an *NRAS* mutation. This could be explained by the existence of an additional alteration on the gene, preventing the specific probe used in dPCR to bind to the expected mutation. The agreement between the two techniques was stronger for *NRAS*-mutated patients than for *BRAF*-mutated patients (Cohen’s κ = 0.83 and 0.64, respectively), precluding any lack of analytical sensitivity of the Cobas technique for the detection of *NRAS* mutations. The total number of gene copies (mutated and wildtype copies, assessed by dPCR) did not differ significantly between *BRAF* and *NRAS*-mutated patients (means: 1.508 *BRAF* copies/mL of plasma vs. 1.893 *NRAS* copies/mL; *p* = 0.115), suggesting that the lower detection of *NRAS* mutations was not linked to a defect in DNA extraction. Finally, ctDNA detectability was not associated with the storage duration of samples before analysis (*p* = 0.193).

Cobas analysis specificity was also evaluated in 20 samples collected from *BRAF* and *NRAS* wildtype patients. The Cobas analysis was negative for all these negative controls, thus, giving a specificity of 100%.

### 2.4. Prognostic Value of CtDNA Detection at First-Line Treatment Initiation

The prognostic value of the detection of circulating DNA related to a previously identified somatic mutation was evaluated in the 57 patients starting first-line systemic therapy (targeted therapy or immunotherapy). The remaining 11 patients, included in the initiation of at least second-line therapy, were studied separately.

**Figure 1:**
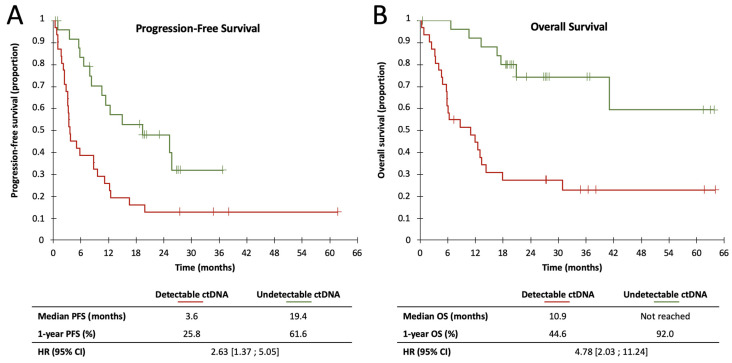
Kaplan–Meier estimates of Progression-Free Survival (**A**) and Overall Survival (**B**) of first-line patients, according to ctDNA detectability.

**Figure 2:**
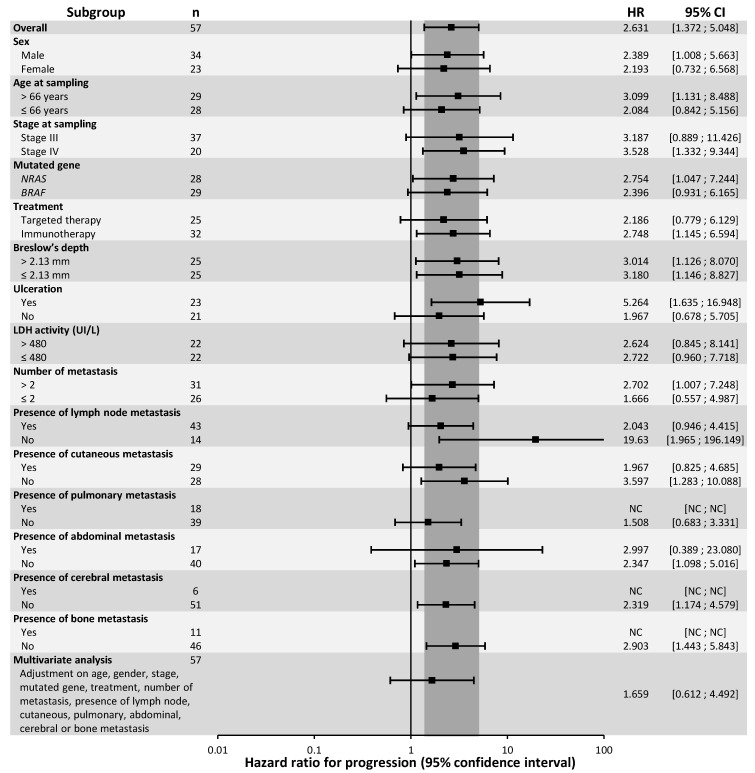
Subgroup and multivariate analysis of Progression-Free Survival, according to ctDNA detectability.

In univariate analysis, ctDNA detection was significantly associated with a poor prognosis for PFS in the first line of treatment (Figure 1; log-rank: *p* = 0.002). Median PFS was 111 days for patients whose ctDNA was detectable, compared to 591 days for patients with undetectable ctDNA at treatment initiation (1-year PFS: 25.8% for detectable ctDNA vs. 61.6% for undetectable ctDNA). Detectable ctDNA was associated with a poor PFS prognosis in immunotherapy-treated patients (median PFS: 101 days for detectable ctDNA vs. 767 days for undetectable ctDNA; log-rank: *p* = 0.019) but was not significantly associated with PFS in targeted therapy-treated patients (median PFS: 144 days for detectable ctDNA vs. 457 days for undetectable ctDNA; log-rank: *p* = 0.128) (Supplementary Figure S1). In subgroup analysis, detection of ctDNA was significantly associated with PFS in only a few subgroups: men; age > 66 years; stage IV melanoma; *NRAS*-mutated melanoma (Figure S2); immunotherapy treated-patients; presence of ulceration; number of metastases > 2; absence of lymph node, skin, abdominal, brain or bone metastases (Figure 2). Detection of ctDNA was not significantly associated with PFS in multivariate analysis, after adjustment for age, sex, stage, mutated gene, nature of treatment, number and nature of metastases (HR = 1.659; CI 95%: (0.612; 4.492); *p* = 0.320).

**Figure 3:**
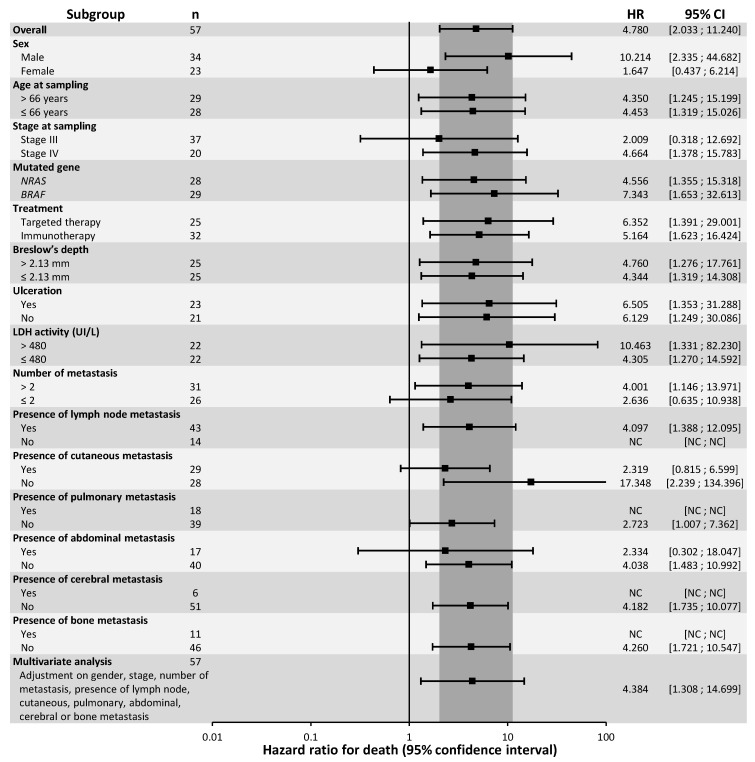
Subgroup and multivariate analysis of Overall Survival, according to ctDNA detectability.

ctDNA detection was strongly associated with poor prognosis of OS in patients on first-line treatment (Figure 1; log-rank: *p* < 0.0001). Detection of ctDNA was associated with a median OS of 332 days and was not reached at the endpoint date for patients whose ctDNA was undetectable (1-year OS: 48.0% for detectable ctDNA vs. 92.0% for undetectable ctDNA). The prognostic value of the detection of ctDNA remained significant, both for patients treated with immunotherapy (median OS: 183 days vs. 1251 days for detectable and undetectable ctDNA, respectively; log-rank: *p* = 0.002) or with targeted therapy (median OS: 379 days vs. not reached for detectable and undetectable ctDNA, respectively; log-rank: *p* = 0.007) (Figure S1). In subgroup analysis, detection of ctDNA was significantly associated with OS in all subgroups except in women, stage III melanoma, patients with a number of metastases ≤ 2, and patients presenting abdominal or skin metastases (Figure 3). ctDNA detection at the first systemic therapy initiation remained a prognostic factor for OS after adjustment for sex, stage, number and nature of metastatic sites in a multivariate analysis (HR = 4.384; 95% CI: (1.308; 14.699); *p* = 0.017).

### 2.5. Prognostic Value of CtDNA Detection at Non-First-Line Treatment Initiation

The prognostic value of detection in circulating DNA of a previously identified somatic mutation was also evaluated in the 11 patients included in initiation of at least a second-line therapy. ctDNA detection was not associated with either PFS or OS at non-first-line treatment initiation, in univariate analysis (log-rank: *p* = 0.054 and 0.763, respectively).

## 3. Discussion 

Our results demonstrate that plasma ctDNA detection at first-line treatment initiation is an independent prognostic factor for OS in AJCC stage IV or unresectable stage III metastatic cutaneous melanoma patients. Several studies have previously shown the negative prognostic value of ctDNA detection for OS in metastatic cutaneous melanoma [12,13,14,15,16]. This is the first study, however, to demonstrate its independent prognostic value, irrespective of treatment, in an intention-to-treat setting. According to a recent study, ctDNA abundance may even be associated with OS regardless of the type of cancer, though its results remain to be confirmed, as this study includes only very few cases of metastatic melanoma [17].

The prognostic value of ctDNA detection for OS is probably linked to its correlation with tumour burden. Indeed, it is now well-established that ctDNA constitutes a highly specific tumour biomarker, quantitatively associated with tumour burden as assessed by CT scan [18], and with tumour metabolism measured by PET scan [19]. These findings are entirely consistent with the correlation observed in this study between ctDNA detection and parameters related to tumour burden, such as the number of metastases or the AJCC stage. Moreover, the release of tumour DNA into the bloodstream also depends on the location of the lesions; brain metastases, in particular, are known to release little DNA into the circulation, due to the blood–brain barrier. This last point may possibly explain why the presence of lymph node, abdominal (including hepatic), pulmonary and bone metastases were associated with better ctDNA detectability in this study, whereas skin and brain metastases were not. 

While tumour burden is likely to condition the patient’s survival and may explain the association between ctDNA detection and OS, ctDNA detection was less correlated with PFS in this study and was not significantly independent from other prognostic factors, despite a clear trend. Larger studies are required to determine whether ctDNA detection is an independent prognostic factor for PFS, particularly in patients treated with immunotherapy, for whom this effect is more pronounced.

ctDNA detectability was also correlated with the nature of the mutated gene; *NRAS* mutations were less frequently identified than *BRAF* mutations in circulating DNA (13/36 patients (36%) vs. 21/32 patients (66%), respectively). The comparison of the Cobas qPCR assay with validated dPCR analysis made it possible to exclude any lack of analytical sensitivity of the Cobas test and was not favourable to a possible defect in the preanalytical procedure (extraction, sample preservation). Moreover, similar trends have been observed in other studies; thus, Long-Mira et al. reported sensitivities of 40% and 67% for *NRAS* and *BRAF* mutations, respectively, with an NGS analysis [20], while Seremet et al. detected ctDNA in 22/46 (48%) and 6/17 (35%) *BRAF* and *NRAS*-mutated patients, respectively, using a dPCR test [16]. A lower ctDNA detectability could be a characteristic of *NRAS*-mutated tumours.

NRAS mutations were more frequent in women than in men, which is consistent with data from our own daily diagnostic activity on tissue DNA. Indeed, out of 3263 samples analysed since 2012 in our routine practice, 822 carried an *NRAS* mutation (25.2%), among which 447/1528 *NRAS*-mutated samples were from women (29.3%) vs. 375/1735 *NRAS*-mutated samples from men (21.6%). ctDNA was also more frequently detected in men than in women, regardless of the nature of the mutated gene. This finding has also been observed in other studies [19,21], though its interpretation remains unclear due to conflicting reports [13,14].

Routine circulating DNA testing is easy and is currently being performed in our hospital for some patients with newly diagnosed metastatic melanoma, to allow a rapid initiation of targeted therapy if a *BRAF* mutation is identified [7]. Indeed, circulating DNA analysis is faster than tumour tissue DNA analysis because plasma does not require the same preanalytical treatment as tumour tissue (formaldehyde fixation, paraffin embedding, making thick tissue sections) [22]. Systematic ctDNA analysis in all metastatic melanoma patients, before the first line of treatment, in addition to tumour tissue DNA analysis, thus, appears feasible and would both accelerate the therapeutic decision and clarify the prognosis of the disease.

The interpretation of the ctDNA prognostic value, however, must take into consideration the existence of a detectable somatic mutation. Indeed, in this study, we highlighted the independent prognostic value of ctDNA detection for OS only in patients with somatic mutations that could be detected by Cobas analysis. Thus, in a routine setting, while the detection of a somatic mutation in circulating DNA would be directly interpreted as an unfavourable prognostic factor for OS (since mutation detection in plasma attests to its presence in tumour tissue), the absence of detection of any somatic mutation in circulating DNA should be interpreted as an undetectable ctDNA and a good prognostic factor of OS only for patients in whom a somatic mutation has been previously identified in tumour tissue DNA. Despite its advantages, circulating DNA analysis, therefore, remains only a complement to tissue DNA analysis and cannot replace it.

The integration of the ctDNA prognostic value into current staging systems has already been suggested. Some authors have proposed upgrading the Tumour, Node and Metastasis staging system (TNM) to the TNMB staging system, by adding a ‘B’ category (for blood), defined by detection (‘B1’) or the absence of detection (‘B0’) of ctDNA, in a similar manner to the ‘M’ category [23]. In the specific case of melanoma, a similar development could be envisaged for the AJCC classification, since it includes the standard anatomical TNM prognostic factors. Such a change would be relevant to guide the therapeutic decision faced with an increasing number of therapeutic options for the management of stage IV metastatic melanoma. For example, the integration of a ‘B’ category for ctDNA would better define which first-line treatment to use in *BRAF*-mutated patients (targeted therapy or first-line immunotherapy), and clarify the choice between anti-PD1 as monotherapy or in combination with anti-CTLA4 in immunotherapy-treated patients. Some studies have also shown ctDNA detection to be an independent prognostic factor for OS in stage III melanoma patients. In this context, an amended AJCC classification would also make it possible to better determine which stage III melanoma patients would be likely to benefit from targeted therapies or immunotherapies, in an adjuvant setting [14], and probably in the near future, in a neoadjuvant setting [24,25]. However, before any integration of ctDNA detection into cancer staging systems, there is an urgent need for an international agreement concerning the analysis of circulating DNA, in order to standardise the methods of analysis, and define their prognostic significance thresholds.

## 4. Material and Methods

### 4.1. Patients and Samples

Plasma samples collected before treatment initiation of stage IV or unresectable stage III melanoma patients at Nantes University Hospital between January 2014 and March 2017 were included in this study. Plasma samples were collected in EDTA tubes (Greiner Bio-One, Les Ulis, France), centrifuged at 2000× *g* for 10 min, and frozen at −80 °C within 4 h after venepuncture.

During this time frame, detection of *BRAF* and *NRAS* mutation in the patient’s tumour was performed using a combination of allele-specific amplification and Sanger sequencing [26,27,28]. Our laboratory is accredited in accordance with the International Standard ISO15189. 

Patients were treated either by immunotherapy (nivolumab monotherapy or nivolumab–ipilimumab combination) or by targeted therapy for *BRAF*-mutated patients (vemurafenib alone or vemurafenib + cobimetinib or dabrafenib + trametinib).

### 4.2. Circulating DNA Analysis

ctDNA was extracted from 2 mL of plasma using the cfDNA Sample Preparation Kit (Roche), and eluted in 100 μL of elution buffer as recommended by the supplier. 

*BRAF* or *NRAS* mutations were screened for with the Cobas BRAF/NRAS Mutation Test LSR kit (Roche), an allele-specific real-time PCR test for the qualitative detection and identification of 36 *BRAF* and *NRAS* mutations. The analysis was carried out according to the supplier’s recommendations. 

The specificity of the Cobas assay was evaluated on 20 negative control plasmas collected from *BRAF* and *NRAS* wildtype patients before treatment initiation. The analytical performance of the Cobas system was also evaluated, at half of the inclusions, by comparison with a digital PCR assay (dPCR) validated in a previous study [26].

### 4.3. Digital PCR 

The QuantStudio 3D Digital PCR System (LifeTechnologies, Illkirch, France) was used. For each sample, a reaction mixture of 15 μL was prepared with 6.5 μL of DNA extract, 7.5 μL of a PCR mix comprising Taq polymerase and deoxynucleotide triphosphates, and 1 μL of a solution containing the primers adapted to the genomic region of interest and two TaqMan probes—one specific to the mutation, labelled with the FAM fluorophore, and the other, specific to the wildtype allele, labelled with the VIC fluorophore (TaqMan dPCR Liquid Biopsy Assays (Thermo Fisher Scientific, Illkirch, France)). References: BRAF476: *BRAF* c.1799A>T (p.V600E); BRAF473: *BRAF* c.1798_1799delinsAA (p.V600K); NRAS564: *NRAS* c.35G>A (p.G12D); NRAS584: *NRAS* c.182A>G (p.Q61R); NRAS580: *NRAS* c.181C>A (p.Q61K); NRAS583: *NRAS* c.182A>T (p.Q61L). This mixture was then partitioned onto a 20,000-well chip by diffusion, using a standardised semi-automatic device. After sealing the chip, the amplification reaction was carried out using a suitable thermal cycler, according to the following program: hold 10 min at 96 °C, followed by 39 cycles alternating for 2 min at 60 °C and 30 s at 98 °C. At the end of the amplification reaction, the fluorescence emitted by each well was read using a dedicated reader; a green FAM fluorescence signal at 518 nm was emitted by the well in the presence of the mutation, while a yellow VIC signal at 554 nm was emitted by the well in the presence of the wildtype allele. These fluorescence data were then analysed using software of our design (unpublished). A sample was considered positive for the mutation when at least 2 wells were positive for the mutation (8 copies/mL of plasma, under the extraction and analysis conditions used in this study).

### 4.4. Response Assessment

Clinical follow-up was performed at each visit. Radiological monitoring was performed by means of a chest, abdomen and pelvis scan and a brain CT scan every 8 weeks. Tumour response was measured according to RECIST v1.1 criteria.

### 4.5. Patient Characteristics

The clinical characteristics of the patients were provided by the National Melanoma Research and Clinical Investigation Database (RIC-Mel network) coordinated by Nantes. These data included age, gender, stage of disease, primary tumour thickness and ulceration, number and location of metastases, baseline LDH activity, and number and nature of previous therapeutic lines.

### 4.6. Statistical Analysis

The endpoint date was set for 16 April 2019. Progression-Free Survival (PFS) was defined as the time between the date of first treatment administration and the first date of documented progression or death for any reason, whichever came first. Patients who had not progressed before the endpoint date were censored on the date of their last assessment. Overall survival (OS) was defined as the time between the date of first treatment administration and death from any cause. Patients who did not die before the endpoint date were censored on the date of their last assessment. Survival probabilities were estimated using the Kaplan–Meier method and compared using a log-rank test. The association between ctDNA detection and OS or PFS was estimated by univariate analysis and by subgroup analysis, using a Cox proportional hazards model and evaluated using the Wald test. Finally, a multivariate analysis with adjustment of the characteristics significantly associated with OS or PFS was performed. 

The Mann–Whitney and Fisher tests were used to compare patient characteristics. All tests were conducted using bilateral assumptions and a significance level p < 0.05 was used to establish the significance of our observations. Statistical analyses of this study were performed using the XLSTAT software programs.

### 4.7. Ethical Aspects

All patients signed a written consent form, authorising blood sampling, storage of the samples in a biocollection and their use for research purposes (biocollection N° DC-2011-1399). The study was conducted in accordance with the Declaration of Helsinki.

## 5. Conclusions

Baseline ctDNA detection upon initiation of a first-line treatment is an unfavourable prognostic factor for overall survival in unresectable stage III or stage IV metastatic cutaneous melanoma patients, independent of other known prognostic factors. Prospective studies comparing their accuracy in predicting melanoma outcomes by AJCC staging to ctDNA-based TNMB staging are critically needed.

## Figures and Tables

**Table 1:** Patient characteristics.

*n*		Total	BRAF-Mutated	NRAS-Mutated	*p*-Value
		68	32	36	
Age (m (Q1–Q3))		62.0 (52.5–72.4)	58.3 (47.3–68.9)	65.3 (61.3–74.2)	0.061
Breslow (m (Q1–Q3))		2.9 (1.4–3.9)	3.2 (1.3–5.0)	2.6 (1.6–3.4)	0.490
Number of metastases (m (Q1–Q3))		3.6 (2–4.3)	3.8 (2.0–5.0)	3.4 (2.0–4.0)	0.442
LDH (m (Q1–Q3))		593.7 (354.6–729.3)	626.8 (372.3–762.9)	561.8 (309.7–649.8)	0.199
Gender	F	31	8 (26%)	23 (74%)	0.002
	M	37	24 (65%)	13 (35%)	
Stage	III	21	8 (38%)	13 (62%)	0.432
	IV	47	24 (51%)	23 (49%)	
Ulceration	No	35	12 (34%)	23 (66%)	0.102
	Yes	33	20 (61%)	13 (39%)	
Presence of lymph node metastasis	No	14	4 (29%)	10 (71%)	0.144
	Yes	54	28 (52%)	26 (48%)	
Presence of cutaneous metastasis	No	33	17 (52%)	16 (48%)	0.627
	Yes	35	15 (43%)	20 (57%)	
Presence of pulmonary metastasis	No	43	19 (44%)	24 (56%)	0.534
	Yes	25	13 (52%)	12 (48%)	
Presence of cerebral metastasis	No	60	27 (45%)	33 (55%)	0.460
	Yes	8	5 (63%)	3 (38%)	
Presence of abdominal metastasis	No	46	21 (46%)	25 (54%)	0.799
	Yes	22	11 (50%)	11 (50%)	
Presence of bone metastasis	No	56	28 (50%)	28 (50%)	0.353
	Yes	12	4 (33%)	8 (67%)	
Treatment	Immunotherapy	43	7 (16%)	36 (84%)	-
	*Anti-PD1*	*35*	*4*	*31*	
	*Anti-CTLA4*	*2*	*1*	*1*	
	*Anti-PD1/anti-CTLA4*	*6*	*2*	*4*	
	Targeted therapy	25	25 (100%)	0 (0%)	
	*BRAFi*	*8*	*8*	*0*	
	*BRAFi + MEKi*	*17*	*17*	*0*	
Therapeutic line	First line	57	29 (51%)	28 (49%)	0.196
	≥second line	11	3 (27%)	8 (73%)	

**Table 2:** Patient characteristics, according to ctDNA detectability in Cobas analysis.

*n*		Total	Undetectable ctDNA	Detectable ctDNA	*p*-Value
		68	34	34	
Age (m (Q1–Q3))		62.0 (52.5–72.4)	62.1 (52.1–73.1)	62.0 (54.7–71.8)	0.598
Breslow (m (Q1–Q3))		2.9 (1.4–3.9)	2.7 (1.3–3.4)	3.0 (1.4–4.0)	0.529
Number of metastases (m (Q1–Q3))		3.6 (2–4.3)	3.3 (2.0–3.0)	3.9 (3.0–5.0)	0.002
LDH (m (Q1–Q3))		593.7 (354.6–729.3)	519.4 (323.4–649.8)	670.8 (391.9–842.6)	0.100
Gender	F	31	22 (71%)	9 (29%)	0.003
	M	37	12 (32%)	25 (68%)	
Stage	III	21	17 (81%)	4 (19%)	0.001
	IV	47	17 (36%)	30 (64%)	
Ulceration	No	35	19 (56%)	15 (44%)	0.414
	Yes	33	15 (44%)	19 (56%)	
Presence of lymph node metastasis	No	14	11 (79%)	3 (21%)	0.033
	Yes	54	23 (43%)	31 (57%)	
Presence of cutaneous metastasis	No	33	15 (45%)	18 (55%)	0.628
	Yes	35	19 (54%)	16 (46%)	
Presence of pulmonary metastasis	No	43	26 (59%)	18 (41%)	0.043
	Yes	25	8 (33%)	16 (67%)	
Presence of cerebral metastasis	No	60	32 (53%)	28 (47%)	0.259
	Yes	8	2 (25%)	6 (75%)	
Presence of abdominal metastasis	No	46	29 (63%)	17 (37%)	0.005
	Yes	22	5 (23%)	17 (77%)	
Presence of bone metastasis	No	56	33 (59%)	23 (41%)	0.003
	Yes	12	1 (8%)	11 (92%)	
Mutated gene	BRAF	32	11 (34%)	21 (66%)	0.028
	NRAS	36	23 (64%)	13 (36%)	
Treatment	Immunotherapy	43	25 (58%)	18 (42%)	0.131
	Targeted therapy	25	9 (36%)	16 (64%)	
Therapeutic line	First line	57	26 (46%)	31 (54%)	0.186
	≥second line	11	8 (73%)	3 (27%)	

**Table 3:** Concordance between Cobas analysis and digital PCR for the first 31 patients included.

		**Digital PCR**	
		**Mutation detected** (BRAF mutations) (NRAS mutations)	**Mutation not detected** (BRAF mutations) (NRAS mutations)
**Cobas qPCR**	**Mutation detected** (BRAF mutations) (NRAS mutations)	18 (10) (8)	2 (1) (1)
	**Mutation not detected** (BRAF mutations) (NRAS mutations)	2 (2) (0)	9 (5) (4)

## Supplementary Materials

The following are available online at https://www.mdpi.com/2072-6694/12/7/1871/s1, Figure S1: Kaplan–Meier estimates of Progression-Free Survival of patients treated in first line with targeted therapy (**A**) or immunotherapy (**B**); Figure S2: Kaplan–Meier estimates of Progression-Free Survival of *BRAF*-mutated patients (**A**) and *NRAS*-mutated patients (**B**).
