# Supplementary material for: Circulating Tumour DNA Is an Independent Prognostic Biomarker for Survival in Metastatic BRAF or NRAS-Mutated Melanoma Patients
Source: Cancers (Basel). 2020 Jul 11;12(7):1871. doi: 10.3390/cancers12071871 (PMC7409003; doi:10.3390/cancers12071871)
Supplement: Supplementary file 1 [file cancers-12-01871-s001.pdf]

Supplementary Materials:

Circulating Tumour DNA is an Independent Prognostic Biomarker for Survival in Metastatic *BRAF* or *NRAS*-Mutated Melanoma Patients

Guillaume Herbreteau, Audrey Vallée, Anne-Chantal Knol, Sandrine Théoleyre, Gaelle Quéreux, Cécile Frénard, Emilie Varey, Paul Hofman, Amir Khammari, Brigitte Dréno and Marc G. Denis

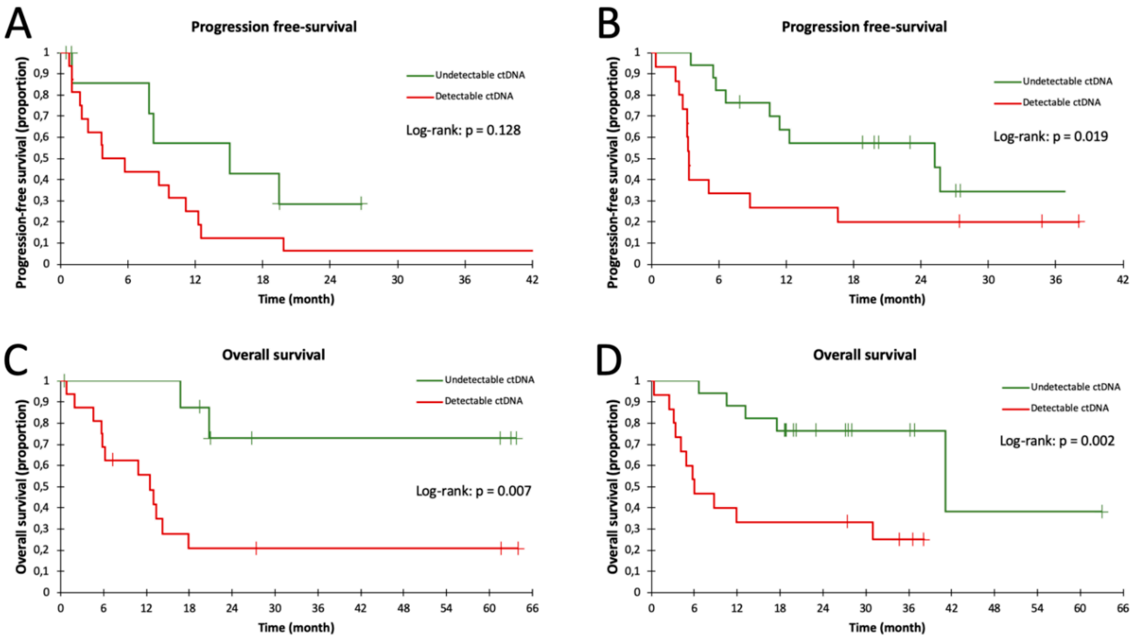

Figure 1. Kaplan-Meier estimates of Progression-Free Survival of patients treated in first line with targeted therapy (**A**) or immunotherapy (**B**). Kaplan-Meier estimates of Overall Survival of patients treated in first line with targeted therapy (**C**) or immunotherapy (**D**).

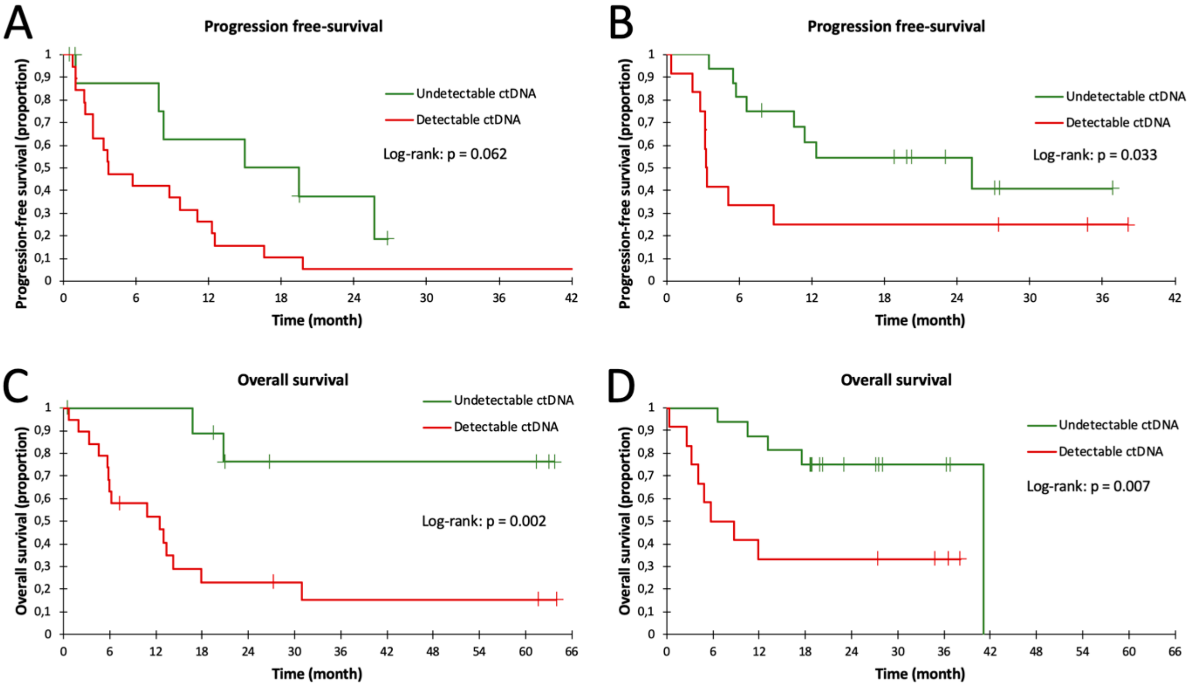

Figure 2. Kaplan-Meier estimates of Progression-Free Survival of *BRAF*-mutated patients (**A**) and *NRAS*-mutated patients (**B**). Kaplan-Meier estimates of Overall Survival of *BRAF*-mutated patients (**C**) and *NRAS*-mutated patients (**D**).
